# Recent Findings of Potentially Lethal Salamander Fungus *Batrachochytrium salamandrivorans*

**DOI:** 10.3201/eid2507.181001

**Published:** 2019-07

**Authors:** David Lastra González, Vojtech Baláž, Milič Solský, Barbora Thumsová, Krzysztof Kolenda, Anna Najbar, Bartłomiej Najbar, Matej Kautman, Petr Chajma, Monika Balogová, Jiří Vojar

**Affiliations:** Czech University of Life Sciences, Prague, Czech Republic (D. Lastra González, M. Solský, B. Thumsová, P. Chajma, J. Vojar);; University of Veterinary and Pharmaceutical Sciences, Brno, Czech Republic (V. Baláž, M. Kautman);; University of Wrocławski, Wroclaw, Poland (K. Kolenda, A. Najbar); University of Zielona Góra, Lubuskie, Poland (B. Najbar);; Slovak Academy of Sciences, Košice, Slovakia (M. Kautman);; Pavol Jozef Šafárik University in Košice, Košice (M. Balogová)

**Keywords:** chytridiomycosis, amphibians, salamanders, newts, caudates, fungi, *Lissotriton helveticus*, *Batrachochytrium salamandrivorans*, Europe

## Abstract

The distribution of the chytrid fungus *Batrachochytrium salamandrivorans* continues to expand in Europe. During 2014–2018, we collected 1,135 samples from salamanders and newts in 6 countries in Europe. We identified 5 cases of *B. salamandrivorans* in a wild population in Spain but none in central Europe or the Balkan Peninsula.

Chytridiomycosis, an amphibian disease caused by the chytrid fungi *Batrachochytrium dendrobatidis* and *B. salamandrivorans*, is responsible for declines of amphibian populations worldwide (*1*). The recently discovered *B. salamandrivorans* (*2*) is severely impacting salamanders and newts in Europe (*3**,**4*). This emerging fungal pathogen infects the skin of caudates and causes lethal lesions (*2*). It most likely was introduced to Europe by the pet salamander trade from Southeast Asia (*3*). In Europe, the Netherlands, Belgium, and Germany have confirmed *B. salamandrivorans* in wild caudates; the United Kingdom, Germany, and Spain have confirmed the fungus in captive animals (*5**,**6*). Several countries have established trade regulations (*5*) and a recent European Union decision, no. 2018/320, implements measures to protect against the spread of *B. salamandrivorans* via traded salamanders (*7*). The World Organisation for Animal Health listed infection with *B. salamandrivorans* as a notifiable disease in 2017. In addition to controlling the amphibian pet trade, surveillance of the pathogen is urgently needed to establish disease intervention strategies in affected areas and prevention in *B. salamandrivorans*–free regions.

During 2014–2018, we collected 1,135 samples directly for the detection of *B. salamandrivorans* or as a part of unrelated studies. Samples came from 10 amphibian species at 47 sites in 6 countries in Europe*.* Most samples came from the fire salamander, *Salamandra salamandra*, which is a known suitable host for *B. salamandrivorans* (*3*), and the palmate newt, *Lissotriton helveticus*, which is known to be resistant to *B. salamandrivorans* (Appendix Table 1).

Most samples were skin swabs collected by following the standard procedure for sampling of amphibian chytrid fungi (*8*). A smaller portion of samples was toe clippings (Appendix Table 2). We extracted genomic DNA following the protocol of Blooi et al. (*9*), and 2 laboratories with different equipment tested for *B. salamandrivorans*. Samples from Spain and the Czech Republic initially were analyzed at the Czech University of Life Sciences (Prague, Czech Republic) by standard PCR with *B. salamandrivorans*–specific primers STerF and STerR, as described by Martel et al. (*2*), with subsequent electrophoresis on the amplified target. We reanalyzed samples that produced positive or equivocal results by using duplex quantitative PCR (qPCR) for *B. dendrobatidis* and *B. salamandrivorans* (*9*) at the University of Veterinary and Pharmaceutical Sciences (Brno, Czech Republic). Trenton Garner of the Institute of Zoology, Zoological Society of London (London, England), provided DNA for quantification standards of the *B. dendrobatidis* GPL lineage, strain IA042, and An Martel of Ghent University (Ghent, Belgium) provided quantification standards of *B. salamandrivorans*. 

We directly analyzed samples from other countries by qPCR. We used negative and positive controls for standard PCR analyses and quantification standards for qPCR analyses. For *B. dendrobatidis*– or *B. salamandrivorans*–positive sites, we estimated prevalence and Bayesian 95% CIs using 3 parallel Markov chains with 2,000 iterations each, a burn-in of 1,000 iterations, and no thinning (Appendix Table 1). We performed all statistical analyses in R 3.3.1 using the R2WinBUGS package and WinBUGS 1.4.3 (*10*).

Samples from 5 *L. helveticus* newts tested positive for *B. salamandrivorans*, implying that this species is not resistant to this fungus as previously indicated by experimental exposures (*3*). The positive cases were found in populations from an isolated area encompassing 2 different regions in northern Spain, Cantabria and Asturias, with remote human populations. Four cases were found in livestock drinking troughs located 150–1,000 m above sea level, and 1 case was found in a pond in a private garden, 30 km from the nearest recorded case. We did not find *B. salamandrivorans*–positive cases in consecutive locations during our monitoring.

Although *B. salamandrivorans* cases have been reported in captive salamanders (*6*), our reported cases were >1,000 km from any area of known *B. salamandrivorans* occurrence (*7*). We also detected *B. dendrobatidis* by duplex qPCR in 11 samples from 3 newt species (*L. helveticus*, *L. vulgaris*, and *Triturus cristatus*) from Spain and Montenegro and 1 captive *Cynops ensicauda* newt from the Czech Republic. The *B. dendrobatidis*–positive cases did not involve co-infection with *B. salamandrivorans*.

We confirmed that the known distribution of *B. salamandrivorans* continues to expand in Europe, indicating that this fungus might be capable of dispersing over long distances (*4*), might be introduced by humans, or might even have been circulating in this geographic range with no detected deaths. Our results should alert the research and conservation community and motivate urgent action to identify regions with early emergence of the disease and implement mitigation measures to prevent further spread of this deadly pathogen.

Appendix Locations, species, origin, type, and number of samples collected during surveillance for *Batrachochytrium dendrobatidis* and *B. salamandrivorans* in Europe during 2014–2018.
