# Supplementary material for: Recent Findings of Potentially Lethal Salamander Fungus Batrachochytrium salamandrivorans
Source: Emerg Infect Dis. 2019 Jul;25(7):1416–8. doi: 10.3201/eid2507.181001 (PMC6590763; doi:10.3201/eid2507.181001)
Supplement: Appendix — Locations, species, origin, type, and number of samples collected during surveillance for Batrachochytrium dendrobatidis and B. salamandrivorans in Europe during 2014–2018. [file 18-1001-Techapp-s1.pdf]

Article DOI: https://doi.org/10.3201/eid2507.181001

Recent Findings of Potentially Lethal Salamander Fungus *Batrachochytrium salamandrivorans*

Appendix

Appendix Table 1. Summary of locations, sample size, findings from *Batrachochytrium dendrobatidis* and *Batrachochytrium salamandrivorans* testing, and prevalence and infection intensities for each*

Location	Species	No.	*Bd*+	*Bsal*+	Bd_{prev} (95% CI)†	$Bsal_{\text{prev}}$ (95% CI)†	*Bd* min–max GE	*Bsal* min–max GE
Czech Republic								
Prague	*Cynops ensicauda*	5	1		0.29 (0.05–0.65)	0.14 (0–0.47)	1.91	
Montenegro								
Moromish	*Lissotriton vulgaris*	35	4		0.10	0.02	0.28–22.25	
	Triturus cristatuts	22	1		(0.04–0.19)	(0–0.07)	1.05	
Liverovići lake	*L. vulgaris*	31	2		0.09 (0.02–0.20)	0.03 (0–0.11)	1.73–1.83	
Spain								
Suances	*L. helveticus*	22		1	‡	0.06		0.42
	T. marmoratus	10				(0.01–0.16)		
Ampuero	*Salamandra salamandra*	9			‡	0.10 (0.01-0.26)		
	L. helveticus	10		1				2.73
Teverga	*L. helveticus*	62		2	‡	0.04		0.89–4.36
	T. marmoratus	11				(0.01–0.09)		
Carracedelo	*L. helveticus*	5	1		‡	0.06	0.54	
	T. marmoratus	11				(0–0.20)		
Ruente	*L. helveticus*	50	2	1	‡	0.04 (0–0.10)	0.24	0.16

**Bd*, *Batrachochytrium dendrobatidis*; Bd_{prev}, prevalence for Bd; *Bsal*, *B. salamandrivorans*; $Bsal_{\text{prev}}$, prevalence for *Bsal*; CI, confidence interval; GE, genomic equivalent; +, positive.
†Calculated using Bayesian probability.
‡Cannot be included because only a subset of the samples were analysed by duplex qPCR.

Appendix Table 2. Locations, species, year, origin, type, and number of samples collected during surveillance for *Batrachochytrium dendrobatidis* and *Batrachochytrium salamandrivorans**

Location	Species	Year	Origin	Sample Type	No.	*Bd*+	*Bsal*+
Croatia							
lokva Majkovi	*Lissotriton vulgaris*	2016	W	S	30		
Crna Mlaka	*L. vulgaris*	2016	W	S	1		
	Salamandra salamandra	2016	W	S	1		
Czech Republic							
Kokořínsko	*L. vulgaris*	2017	W	S	44		
Ústí nad labem	*S. salamandra*	2016	W	S	17		
Prague	*Cynops ensicauda*	2017	C	S	5	1	
Montenegro							
Moromish	*L. vulgaris*	2016	W	S	35	4	
	Triturus cristatuts	2016	W	S	22	1	
Liverovići lake	*L. vulgaris*	2016	W	S	31	2	
Lovcén	*L. vulgaris*	2016	W	S	40		
Traktir-Sutorina	*L. vulgaris*	2016	W	S	33		
		2016	W	S	10		
Poland							

Location	Species	Year	Origin	Sample Type	No.	*Bd*+	*Bsal*+
Wąwóz Lipa-Chełmy Landscape Park	*S. salamandra*	2014	W	S	30		
Sady-Ślęża Massif	*S. salamandra*	2014	W	S	9		
		2015	W	S	2		
Złoty Stok-Śnieżnik Landscape Park	*S. salamandra*	2014	W	S	15		
		2015	W	S	3		
Jarnołtówek	*S. salamandra*	2015	W	TC	21		
Bielsko-Biała	*S. salamandra*	2016	W	S	32		
Pleśna	*S. salamandra*	2014	W	TC	4		
		2016	W	TC	30		
Góra Kamińska	*S. salamandra*	2015	W	TC	17		
		2016	W	TC	30		
Rakówka	*S. salamandra*	2015	W	TC	7		
Czarnorzeki	*S. salamandra*	2015	W	TC	24		
Trzciana	*S. salamandra*	2014	W	TC	2		
		2016	W	TC	30		
Sękowiec	*S. salamandra*	2016	W	TC	30		
Southern Otryt	*S. salamandra*	2016	W	TC	18		
Jagiellonian University	*L. vulgaris*	2016	C	S	5		
Slovakia							
Remetské Hámre	*S. salamandra*	2017	W	S	15		
Ruská Bystrá	*S. salamandra*	2017	W	S	10		
Tichá Voda	*S. salamandra*	2017	W	S	18		
Ružín	*S. salamandra*	2017	W	S	5		
Modra	*S. salamandra*	2017	W	S	5		
Pezinok	*S. salamandra*	2018	W	S	12		
		2017	W	S	13		
Bratislava	*S. salamandra*	2018	W	S	13		
Spain							
Boo de Guarnizo	*Lissotriton helveticus*	2017	W	S	28		
Santillana del Mar	*Ichthyosaura alpestris*	2017	W	S	10		
	L. helveticus	2017	W	S	1		
	Ambystoma mexicanum	2017	C	S	1		
Suances	*L. helveticus*	2017	W	S	22		1
	Triturus marmoratus	2017	W	S	10		
Valdáliga	*L. helveticus*	2017	W	S	17		
	I. alpestris	2017	W	S	4		
Voto	*S. salamandra*	2017	W	S	19		
Ampuero	*S. salamandra*	2017	W	S	9		
	L. helveticus	2017	W	S	10		1
Teverga	*L. helveticus*	2017	W	S	62		2
	T. marmoratus	2017	W	S	11		
Villafranca del Bierzo	*Lissotriton boscai*	2017	W	S	20		
Carucedo	*L. boscai*	2017	W	S	4		
	L. helveticus	2017	W	S	20		
Carracedelo	*L. helveticus*	2017	W	S	5	1	
	T. marmoratus	2017	W	S	11		
Chozas de Abajo	*Pleurodeles waltl*	2017	W	S	17		
	T. marmoratus	2017	W	S	1		
Ruente	*L. helveticus*	2017	W	S	50	2	1
Cabuérniga	*S. salamandra*	2017	W	S	19		
Campoo-Cabuérniga	*L. helveticus*	2017	W	S	11		
	I. alpestris	2017	W	S	2		
Los Tojos	*T. marmoratus*	2017	W	S	14		
	L. helveticus	2017	W	S	15		
Comillas	*L. helveticus*	2017	W	S	28		
Campoo de Suso	*L. helveticus*	2017	W	S	7		
	I. alpestris	2017	W	S	16		

**Bd*, *Batrachochytrium dendrobatidis*; *Bsal = B.salamandrivorans*; C, captive; S, swab; TC, toe clipping; W, wild; +, positive.
